# Brain network and activity in frontal regions change in the process of visuomotor adaptation

**DOI:** 10.1038/s41598-025-27589-3

**Published:** 2025-12-11

**Authors:** Saki Niiyama, Rie Yoshida, Megumi Miyashita, Toshiyuki Kondo

**Affiliations:** Tokyo University of Agriculture and Technology, Computer and Information Sciences, Koganei, 184-8588 Japan

**Keywords:** Visuomotor task, Near-infrared spectroscopy, Effective connectivity, Neuroscience, Health care, Mathematics and computing

## Abstract

Frontal regions are involved in early stages of motor adaptation. However, the interactions between prefrontal cortex (PFC) and motor cortex (MC) remain unclear. Therefore, we investigated the relationship between PFC and MC in the process of motor adaptation including the early stage, using analyses of brain activity and its effective connectivity (EC). To promote motor adaptation, right-handed participants were trained to perform a visuomotor task with their right hand. To investigate the involved brain networks, their brain activity before and after training was measured by near-infrared spectroscopy (NIRS). EC analysis revealed that the brain network connecting the right PFC and contralateral (left) MC attenuated after the visuomotor training. Brain activity analysis showed the significant decrease in the ipsilateral (right) primary motor cortex (M1) after the visuomotor training. These results suggest that the PFC and MC are synchronized at the early stages of motor learning but act independently through the motor adaptation.

## Introduction

Visuomotor learning refers to the process of coordinating visual input and motor actions to produce suitable movements. So far, motor learning, including visuomotor learning, has been investigated through two main paradigms: motor sequence learning and motor adaptation^1–3^. The former optimizes a particular order of action to best execute a behavior while the latter refers to the ability of kinematically or kinetically adjusting the response to a change in the environment. Particularly, adaptation in visuomotor learning acquires spatial information to compensate for the change. In practice, both motor learning modes probably occur simultaneously, although to different degrees. At the early stages of motor learning, motor sequence learning and motor adaptation seem to recruit similar cerebral structures: the striatum, cerebellum, motor cortical regions, as well as prefrontal and parietal areas^4^. Given the focus on motor sequence learning over the past century^2^, there are fewer studies on motor adaptation than on motor sequence learning.

Despite the cerebellum’s critical role in motor adaptation^5–8^, the cortex, particularly the frontal region, is also involved in the early stage of motor adaptation. In this region, activity in the prefrontal cortex (PFC) increases in the early stage of visuomotor adaptation^9–12^, and activity related to visuomotor task transforms from the pre-supplementary motor area (pre-SMA) to the supplementary motor area (SMA) through motor adaptation^13^. In addition, research on the interactions between the cerebellum and the frontal region suggest modulated connectivity of the cerebellum to the dorsal premotor cortex (dPMC), leading to increased dPMC activation through visuomotor adaptation^14^. Thus, brain activity of the specific frontal region was investigated extremely, but their interactions within the frontal region, which is the brain networks between the PFC and the motor cortex (MC), are still unclear.

Near-infrared spectroscopy (NIRS) is an optical neuroimaging method that allows functional measurement of the cortex, including the frontal region, without local restrictions^15,16^. As one of the NIRS analysis methods, brain activity is well known, but connectivity analysis is also used^17,18^. Connectivity analysis is classified into functional connectivity (FC) and effective connectivity (EC). In contrast to the FC, EC represents the directional connectivity between two nodes and allows analyzing the interaction between the PFC and MC. The EC has been used for the analysis of NIRS data^19–22^. Comparing the average number of EC, which represents the average degree in graph theory, in the PFC and MC before and after motor learning is analogous to estimate of a network change between the PFC and MC.

Therefore, we investigated how the networks connecting the PFC and MC are changed by motor adaptation. To this end, participants performed a visuomotor task in which they used their dominant hand (right hand) to follow a moving target on a computer screen with the cursor, before and after visuomotor training. The task consisted of 30 trials, both behavior and NIRS data were measured during all trials both before (pre-test) and after (post-test). The mean absolute error (MAE) between the target and cursor was calculated from the behavioral data, while the NIRS data were separated into four regions: left prefrontal region (LF), right prefrontal region (RF), left motor region (LM), and right motor region (RM); the average degree and brain activity in these four regions were analyzed as well. By comparing those the obtained results between the pre-test and post-test, we investigated the relation of brain networks (PFC-MC) and MAE.

## Results

### Behavioral data

**Fig. 1:**
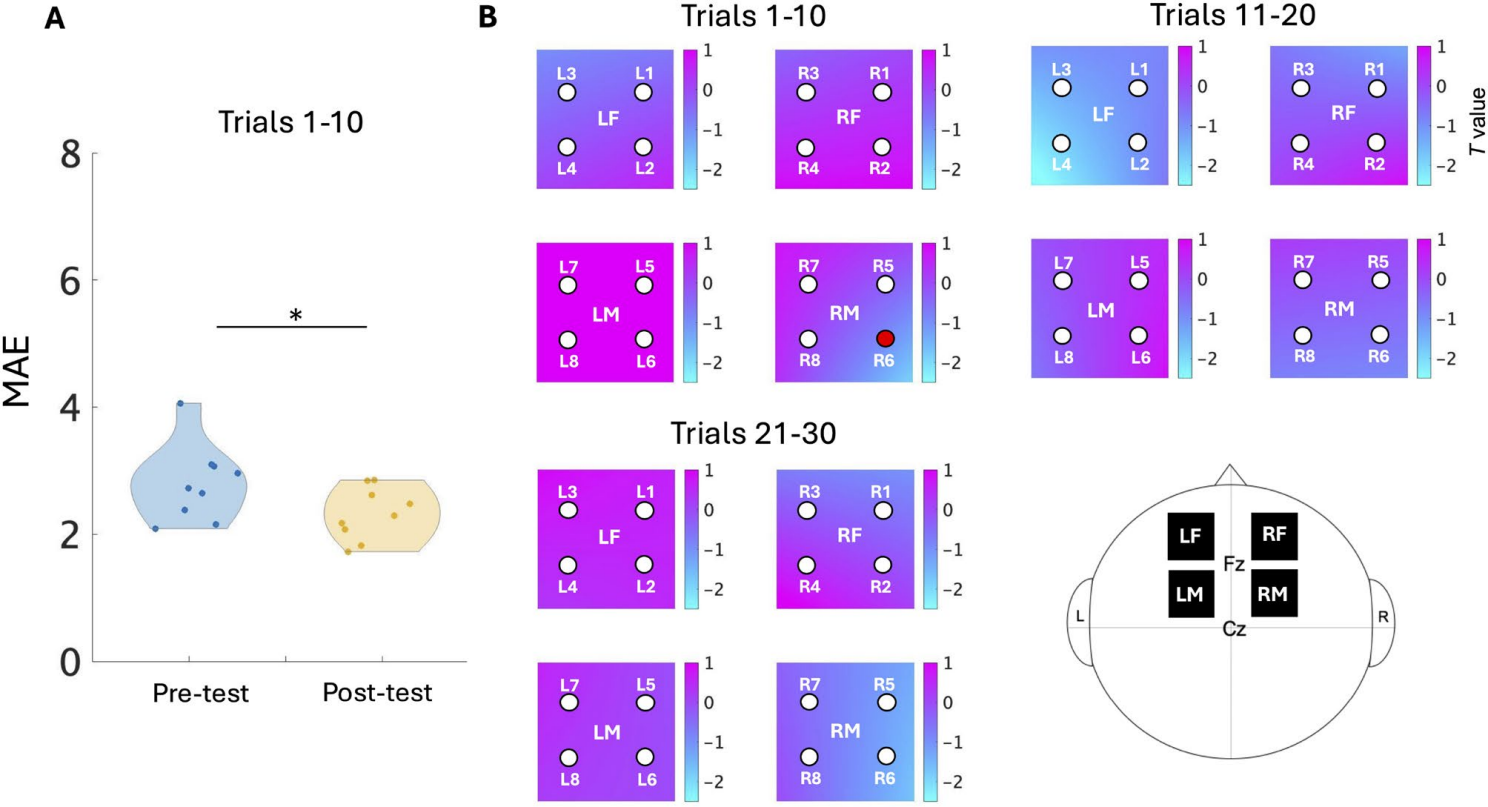
Comparison of behavioral data and the contrast of brain activity between post-test and pre-test. (**A**) Violin plots of mean absolute error (MAE) in the first 10 trials ($n=9$, $^*p <0.05$). Blue and yellow indicate pre-test and post-test, respectively. (**B**) Color maps created from *t* values ($n=9$). High *t* values indicate strong brain activity of post-test compared to pre-test. On color maps, dots indicate the channels measured by NIRS. A red dot indicates a channel showing a significant change between post-test and pre-test ($p <0.05$).

Table 1 shows results of comparison between pre-test and post-test in each trial block (trials 1-10, 11-20, or 21-30) using Wilcoxon signed-rank test. In trials 1-10, the MAE of post-test decreased significantly compared to pre-test ($p(W)=0.02(42)$), and the effect size between both tests was large ($d=0.93$). There were no significant decrease at post-test, compared to at pre-test in trials 11-20 and 21-30, but decreases were observed with small effect sizes between both tests (trials 11–20: $d=0.22$, trials 21–30: $d=0.26$). Figure 1A shows the violin plots of MAE at pre-test and post-test in first 10 trials. This indicates that motor skills improved with the motor training we have constructed.

**Table 1:** Behavioral results.

	*p* (*W*)	*d*	Pre-test	Post-test
			Mean ± SD	
Trials 1–10	0.02 (42)$^{*}$	0.93	$2.80 \pm 0.60$	$2.32 \pm 0.41$
Trials 11–20	0.25 (33)	0.22	$2.80 \pm 0.63$	$2.62 \pm 1.04$
Trials 21–30	0.25 (33)	0.26	$2.89 \pm 0.77$	$2.67 \pm 0.89$

Results of mean absolute error (MAE) at pre-test vs. post-test in each trial block ($n=9$). The *p* values were calculated using Wilcoxon signed-rank test, and values in bracket represent statistics, *W* ($^*p< 0.05$). The *d* represents the Cohen’s effect size. The Mean ± SD represents averaged MAE and its standard deviation across nine participants.

### Brain activity

Table 2 indicates results of two-sample Wilcoxon signed-rank tests (post-test vs. pre-test in each trial block). The color maps in Fig. 1B represent *t* values of *t*-tests for the contrast between post-test and pre-test in each trial block, which indicates how much brain activity of post-test varies relative to pre-test (baseline). In trials 1–10, the brain activity in channel R6 (RM) decreased significantly at post-test compared to pre-test ($p(W)=0.02(3)$) and the medium effect was observed ($d=-0.57$). Increases in channel L5, L6, L7, and L8 (LM) at post-test were observed compared to pre-test, with the medium to large effect size (L5: $d=0.54$, L6: $d=0.73$, L7: $d=0.69$, L8: $d=1.01$). There were no significant change between post-test and pre-test in trials 11–20 and 21–30. However, brain activity in channel L4 (LF) and R1 (RF) decreased at post-test compared to pre-test in trials 11–20 with the medium effect size (L4: $d=-0.75$, R1: $d=-0.50$). At post-test in trials 21-30, decreased activities in channel R5 and R6 (RM) were observed with the medium to large effect sizes (R5: $d=-0.67$, R6: $d=-0.94$).

**Table 2:** Results of brain activity.

	Channel	Trials 1–10				Trials 11–20				Trials 21–30			
		Mean	SD	*p*(*W*)	*d*	Mean	SD	*p*(*W*)	*d*	Mean	SD	*p*(*W*)	*d*
LF	L1	– 21.84	96.53	0.82 (20)	– 0.31	– 44.55	157.50	0.50 (16)	– 0.40	30.86	155.69	0.57 (28)	0.24
	L2	27.26	134.68	0.25 (33)	0.25	– 21.12	96.04	0.57 (17)	– 0.27	18.68	108.22	1.00 (22)	0.31
	L3	– 34.93	108.02	0.30 (13)	– 0.40	– 192.52	557.08	0.82 (20)	– 0.48	41.14	136.39	0.57 (28)	0.40
	L4	2.42	113.53	0.82 (25)	0.03	– 54.68	59.88	0.05 (6)	– 0.75	9.94	105.23	0.65 (18)	0.13
LM	L5	51.70	145.76	0.13 (36)	0.54	28.70	183.81	0.73 (26)	0.17	– 3.36	106.02	0.82 (25)	– 0.04
	L6	43.28	92.56	0.25 (33)	0.73	49.09	169.87	0.50 (29)	0.31	– 1.95	154.28	0.82 (20)	– 0.02
	L7	116.74	233.42	0.10 (37)	0.69	– 5.50	115.40	0.82 (20)	– 0.06	29.74	156.48	0.91 (24)	0.24
	L8	87.68	136.13	0.13 (36)	1.01	– 25.49	118.27	0.57 (17)	– 0.30	10.90	178.90	0.65 (18)	0.08
RF	R1	1.29	130.70	0.50 (29)	0.01	– 35.95	80.96	0.25 (12)	– 0.50	– 41.70	120.36	0.43 (15)	– 0.38
	R2	132.71	430.61	0.82 (20)	0.43	20.62	68.98	0.91 (24)	0.25	5.93	134.80	0.65 (18)	0.05
	R3	– 9.42	69.16	0.65 (18)	– 0.16	– 19.06	90.28	0.50 (29)	– 0.29	– 19.57	79.92	0.57 (17)	– 0.26
	R4	156.16	528.78	0.43 (15)	0.42	1.51	64.36	0.91 (21)	0.03	18.74	48.82	0.25 (33)	0.46
RM	R5	3.24	134.82	1.00 (22)	0.03	1.72	84.51	0.65 (27)	0.03	– 56.62	126.99	0.50 (16)	– 0.67
	R6	– 48.33	74.34	0.02 (3)$^*$	– 0.57	– 47.69	155.96	1.00 (22)	– 0.42	– 62.79	108.96	0.16 (10)	– 0.94
	R7	32.49	119.28	0.91 (24)	0.39	4.00	55.38	0.57 (28)	0.11	– 7.25	63.75	0.65 (27)	– 0.17
	R8	8.05	58.74	0.91 (21)	0.20	– 15.06	58.51	0.82 (25)	– 0.34	– 0.61	54.18	0.65 (27)	– 0.02

The comparison of brain activity at post-test vs. pre-test in each trial block ($n=9$). The *p* was calculated using Wilcoxon signed-rank test, and the *W* represent statistics ($*p < 0.05$ ). The *d* represents the Cohen’s effect size. The mean and standard deviation represent the mean difference in the average integrated value of oxygenated hemoglobin (oxy-Hb) per second (post-test minus pre-test) and their standard deviations, respectively, for nine subjects.

### Effective connectivity

**Fig. 2:**
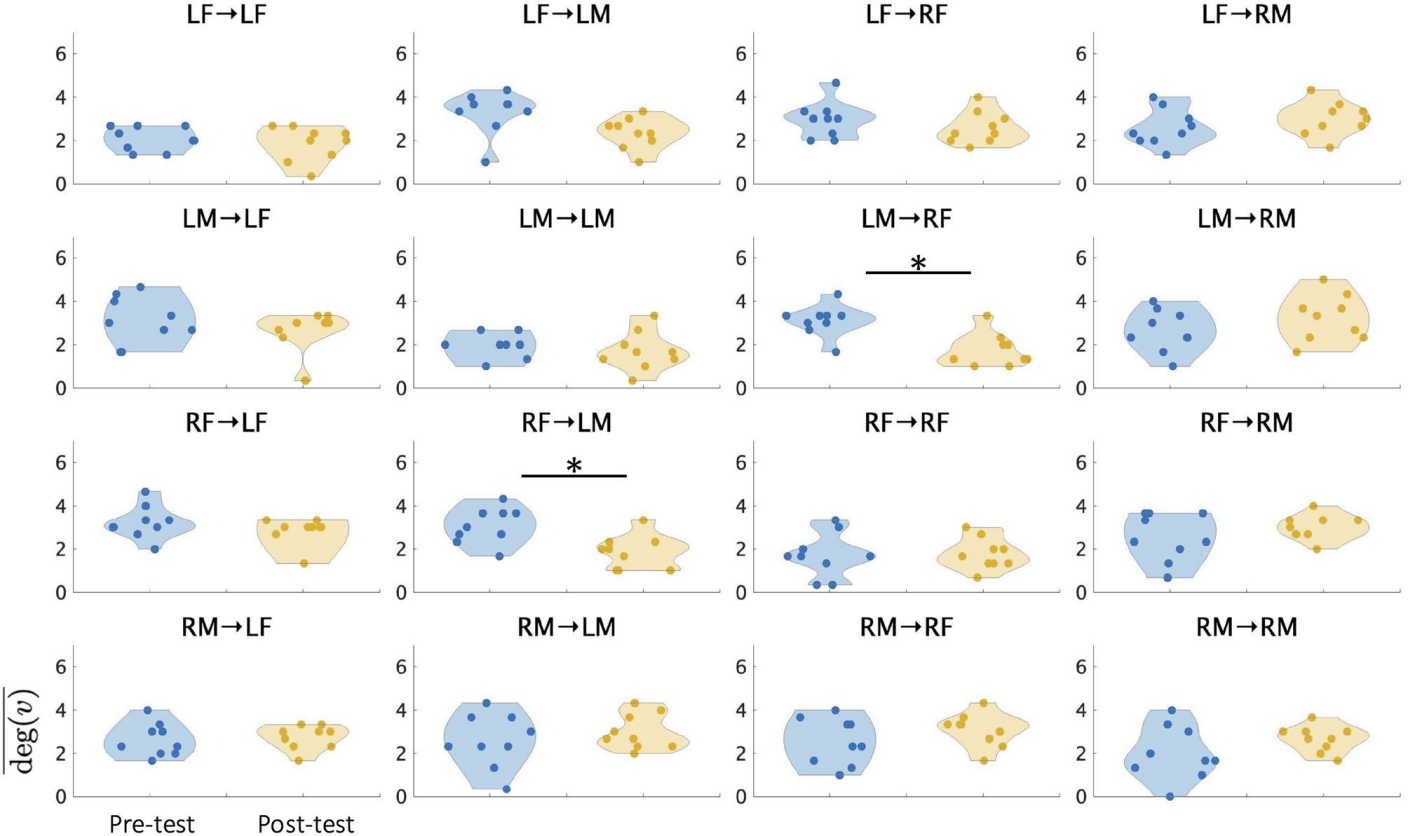
Violin plots of 16 $\overline{\textrm{deg}(v)}$ at pre-tests and post-tests in the trials 21–30 ($n=9$, $^*p<0.05$). Each $\overline{\textrm{deg}(v)}$ represents average degree among four regions (LF, RF, LM, and RM). Blue and yellow plots represent pre-tests and post-tests, respectively. The greater the $\overline{\textrm{deg}(v)}$, the higher the increase in the number of brain networks.

The average degree is represented as $\overline{\textrm{deg}(v)}$. Table 3 shows the *p* value of Wilcoxon signed-rank test and Cohen’s *d* of $\overline{\textrm{deg}(v)}$ compared post-test to pre-test in each trial block. In trials 1–10, there was no significant change between pre-test and post-test, and only small effect sizes were observed. In trials 11–20, the decrease of RF$\rightarrow$RM showed the medium effect size, at post-test compared to pre-test (RF$\rightarrow$RM: $d=-0.52$), but significant changes were not observed. In trials 21–30, RF$\rightarrow$LM and LM$\rightarrow$RF showed significant difference between pre-test and post-test (RF$\rightarrow$LM: $p(W)=0.02(41.5)$, LM$\rightarrow$RF: $p(W)=0.02(34.5)$). In effect size of trials 21–30, RF$\rightarrow$LM and LM$\rightarrow$RF observed the large effect size (RF$\rightarrow$LM: $d=1.01$, LM$\rightarrow$RF: $d=1.25$), and LF$\rightarrow$LM showed the medium size effect (LF$\rightarrow$LM: $d=0.72$). Figure 2 shows the comparisons of $\overline{\textrm{deg}(v)}$ during pre-tests and post-tests in four regions. Using each 16 $\overline{\textrm{deg}(v)}$, which indicates the average number of EC between the four regions (LF, RF, LM, and RM), we investigated the variations in $\overline{\textrm{deg}(v)}$ between pre-tests and post-tests in last 10 trials using Wilcoxon signed-rank tests. In LM$\rightarrow$RF and RF$\rightarrow$LM, which showed significant differences between pre-test and post-test, the $\overline{\textrm{deg}(v)}$ of post-test significantly decreased compared to pre-test.

**Table 3:** Results of effective connectivity.

Region		Trials 1–10						Trials 11–20						Trials 21–30					
		Pre-test		Post-test		*p*(*W*)	*d*	Pre-test		Post-test		*p*(*W*)	*d*	Pre-test		Post-test		*p*(*W*)	*d*
Source	Sink	Mean	SD	Mean	SD			Mean	SD	Mean	SD			Mean	SD	Mean	SD		
LF	$\rightarrow$ LF	2.19	1.00	2.15	0.53	0.91 (19.5)	0.03	2.19	0.85	1.93	0.62	0.15 (28.0)	0.27	2.07	0.55	1.85	0.80	0.75 (26.0)	0.19
	$\rightarrow$ LM	2.81	0.99	2.96	0.63	0.70 (15.0)	– 0.14	2.81	0.67	3.11	0.91	0.55 (13.0)	– 0.21	3.30	0.98	2.33	0.71	0.08 (25.0)	0.72
	$\rightarrow$ RF	2.70	0.79	2.70	0.72	0.95 (23.5)	0.00	2.67	1.20	2.63	0.96	0.93 (23.5)	0.02	2.96	0.82	2.59	0.74	0.45 (24.0)	0.27
	$\rightarrow$ RM	2.63	0.89	2.74	0.55	0.65 (18.0)	– 0.11	2.85	0.94	3.22	0.97	0.54 (16.5)	– 0.22	2.59	0.85	3.00	0.78	0.41 (11.5)	– 0.26
LM	$\rightarrow$ LF	2.70	1.25	2.93	0.70	0.76 (19.5)	– 0.13	2.63	0.65	2.85	0.50	0.59 (13.5)	– 0.27	3.11	1.08	2.63	0.92	0.63 (13.5)	0.31
	$\rightarrow$ LM	2.52	0.60	1.96	0.90	0.31 (16.0)	0.40	1.96	0.61	2.33	1.04	0.48 (16.0)	– 0.26	1.96	0.54	1.70	0.89	0.44 (19.0)	0.21
	$\rightarrow$ RF	3.11	0.85	2.70	0.54	0.30 (26)	0.39	2.70	0.54	2.78	0.78	0.91 (9.50)	– 0.07	3.11	0.71	1.74	0.76	0.02$^*$ (34.5)	1.25
	$\rightarrow$ RM	3.15	0.93	2.63	0.73	0.42 (19.5)	0.44	2.74	0.70	3.04	0.90	0.53 (13.0)	– 0.23	2.63	0.96	3.22	1.07	0.35 (11.0)	– 0.35
RF	$\rightarrow$ LF	2.74	0.72	2.67	0.93	0.76 (20.0)	0.11	2.44	0.82	2.41	1.12	1.00 (14.0)	0.02	3.22	0.76	2.85	0.60	0.72 (13.0)	0.29
	$\rightarrow$ LM	2.63	0.89	2.81	0.38	0.63 (14.0)	– 0.20	2.81	0.44	2.48	0.97	0.34 (31.0)	0.29	3.07	0.83	1.85	0.78	0.02$^*$ (41.5)	1.01
	$\rightarrow$ RF	1.89	0.99	2.19	0.99	0.63 (18.0)	– 0.17	2.26	0.88	1.93	0.66	0.48 (23.5)	0.27	1.70	1.02	1.78	0.73	0.82 (16.0)	– 0.05
	$\rightarrow$ RM	2.81	1.24	3.15	0.60	0.48 (9.5)	– 0.28	2.74	0.83	3.41	0.97	0.16 (10.0)	– 0.52	2.56	1.11	3.00	0.58	0.51 (16.5)	– 0.29
RM	$\rightarrow$ LF	2.78	1.01	2.89	0.71	0.96 (17.0)	– 0.09	2.67	0.88	3.22	0.85	0.37 (14.5)	– 0.34	2.63	0.75	2.74	0.55	0.62 (18.0)	– 0.10
	$\rightarrow$ LM	3.44	1.18	3.19	0.65	0.65 (22.0)	0.18	2.89	0.53	3.04	0.99	0.57 (13.5)	– 0.15	2.59	1.24	3.00	0.82	0.84 (20.5)	– 0.22
	$\rightarrow$ RF	2.81	1.03	2.59	0.88	0.50 (29.0)	0.15	3.04	0.75	3.52	0.94	0.27 (12.5)	– 0.39	2.56	1.08	3.07	0.78	0.48 (12.5)	– 0.33
	$\rightarrow$ RM	2.00	0.82	2.30	0.86	0.63 (11.0)	– 0.24	2.52	0.87	2.67	0.58	0.66 (14.5)	– 0.18	2.00	1.25	2.67	0.60	0.17 (10.5)	– 0.53

Average degree, $\overline{\textrm{deg}(v)}$ at pre-test vs. post-test in each trial block ($n=9$). The *p* values were calculated by Wilcoxon signed-rank test, and values in bracket represent statistics, *W* ($*p< 0.05$). The *d* value represents Cohen’s effect size. Mean and SD represent the value averaged across $\overline{\textrm{deg}(v)}$ of nine participants and its standard deviation.

## Discussion

**Fig. 3:**
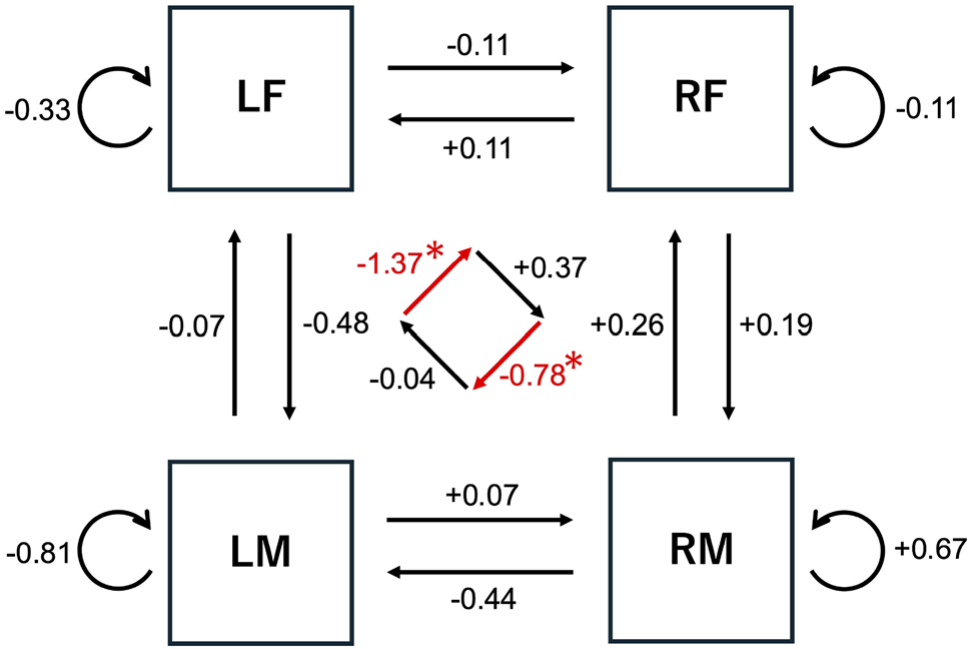
Schema of EC among four regions (LF, RF, LM, and RM). Each value shows average $\overline{\textrm{deg}(v)}$ variation between post-test and pre-test, which represents mean change of average degree across analyzed participants ($n=9$). The red arrows indicate the effective connectivity (EC) showing significant decreases of post-test compared to pre-test ($^*p<0.05$), whereas black arrows indicate EC showing no significant variations.

Our main finding is that the brain networks between the right PFC and the contralateral (left) MC significantly decreased (Fig. 3) and brain activity in the ipsilateral primary motor cortex (M1) significantly decreased, through visuomotor learning.

### Synchronization between the right PFC and the contralateral MC during visuomotor learning

The right PFC, especially the dorsolateral prefrontal cortex (DLPFC), is involved in early stages of visuomotor adaptation^23^ to learn the association between visual and motor commands^24^. Working memory, short-term memory storage, is also required to perform tasks^25,26^. Spatial working memory, not non-spatial memory, is associated with visuomotor tasks, and activation of right DLPFC is greater during a spatial working memory task^27,28^. In the MC, M1 controls the contralateral side of the body commonly due to pyramidal decussation^29^. Thus, the result of EC analysis suggests synchronization between the right PFC and the contralateral MC during visuomotor learning at early stages to transfer the information from the spatial working memory and motor function. Regarding motor sequence learning, a previous study using transcranial magnetic stimulation observed disinhibitted interactions (left DLPFC-contralateral M1) after motor sequence learning with the right hand^30,31^. As motor sequence learning rather requires non-spatial memory, the result is likely consistent with the results of the aforementioned study. However, the measured regions of the prefrontal cortex and the motor cortex contain pre-SMA and PMC/SMA, respectively, so it is possible that these areas (i.e., pre-SMA, PMC/SMA) involve synchronization.

### Decreased brain activity in the ipsilateral MC and inhibitory interhemispheric interaction

During right hand movement, activity in the contralateral MC, including the PMC and M1, increases^32,33^. Unlike the contralateral MC, the decreased activation (deactivation) is observed in the ipsilateral MC, especially the M1^34,35^. Deactivation is associated with inhibitory interhemispheric interaction^35,36^. The brain activities, which showed activation of the contralateral MC and the deactivation of the ipsilateral MC, suggest that an inhibitory interaction from contralateral to ipsilateral MC with motor learning. Although the result of the brain activity analysis was consistent with previous reports, no interhemispheric interaction was observed in the EC analysis. In this study, we investigated changes in the average number of EC, corresponding to the number of brain networks before and after motor learning, but could not specify whether inhibitory or excitatory interaction affected the brain activity, due to the properties of this analysis method. In addition, the changes in the networks indicate the existence of inhibition or excitation, but the lack of variation in the networks does not imply absence of inhibition or excitation. Therefore, we consider that there was inhibitory interhemispheric interaction, despite the lack of change in the networks.

### Limitations and future research directions

Although, based on our results, interactions in the frontal regions related to visuomotor learning were narrowed down to two interhemispheric ones (PFC-MC, MC-MC), we could not pinpoint specific regions (e.g., DLPFC, pre-SMA, SMA, PMC, M1) as we identified cerebral locations based on the international 10–20 system. In addition, we could not verify whether the interaction between the PFC and MC is inhibitory or excitatory. In the future, we need to specify the regions captured during the measurement of NIRS and identify whether inhibitory or excitatory interaction is involved using analysis techniques such as dynamic causal modeling.

## Methods

### Participants

Participants were selected among college students with normal or corrected normal vision (age: 22.0 ± 1.8 [mean ± standard deviation] years). Ten individuals (male: 4, female: 6) were recruited, all right-handed as classified by the Edinburgh Handedness Inventory. Although 10 participants are a small sample size, a previous study in motor learning demonstrated that the activity in the frontal polar cortex decreased through motor training at post-training compared to pre-training with six participants^37^. Therefore, we decided on 10 participants as a sample size in this study. This study was carried out following the ethical principles of the Declaration of Helsinki, under the approval of the Ethics Committee of Tokyo University of Agriculture and Technology (Approval No. 231005-0548). All participants were provided informed consent before beginning with the experiments.

### Visuomotor task

As a motor learning task, we developed a visual tracking task using a haptic device (Touch X, 3D Systems, South Carolina, USA). In it, participants were asked to follow a target moving along an unpredictable trajectory on a one-dimensional (horizontal) axis using a hand cursor with the device. To develop the visuomotor task, we first designed the target motion to be tracked. The desired trajectory of the target *y*(*t*) was determined based on the following equation:

1 $$\begin{aligned} y(t) = 0.64\sin (1.8t) + 0.25\sin (1.82t) + 0.43\sin (2.34t){.} \end{aligned}$$

**Fig. 4:**
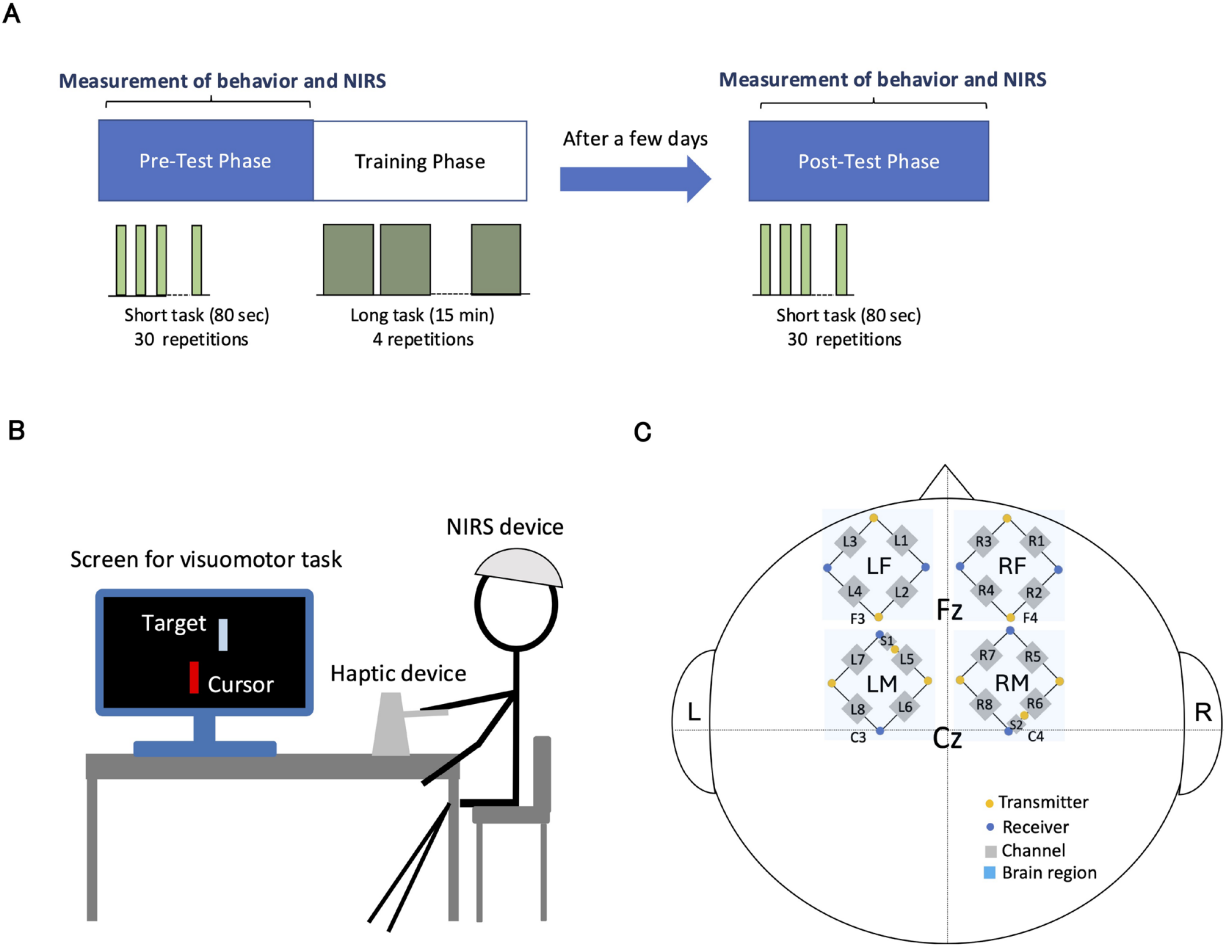
Experimental design. (**A**) Experimental procedure. (**B**) Experimental setup during phases of pre-test and post-test. (**C**) Arrangement of optodes and channels in the NIRS device.

This equation was designed based on a previous study^38^. The visible target was controlled to follow the desired trajectory by the arbitrary feedback system which governs the degree of coincidence between the desired and actual target positions , so that participants learn the task sufficiently through practice. The target appeared on the screen as a light blue bar. The participants handled the haptic device and operated a red bar as a cursor on the screen, as shown in Fig. 4B. The motion range of the haptic device was constrained in the horizontal axis, so they could only move the cursor horizontally. Participants were asked to continue following the moving target to match the positions of the target and cursor horizontally during the task period. When the target and cursor were in the same horizontal position, the cursor color changed to light blue (same color as the target). This allowed them to understand when the position of the cursor exactly matched the target. During the visuomotor task, participants operated the haptic device using their right hand.

### Experimental schedule

As shown in Fig. 4A, the experiment comprised three phases: pre-test, training, and post-test. From our preliminary research, we knew that consecutive execution of all phases in a single day caused low brain activity due to fatigue and that the effect of motor learning is maintained for a few days. Moreover, previous studies indicate that either sleep or passage of time has a effect of consolidation of motor adaptation^39–41^. Therefore, we set the post-test phase a few days after the pre-test and training phases. The day range between the pre-test and training phase and the post-test phase was 2 or 3 days (mean ± standard deviation: 2.6 ± 0.5, median: 3, $n=10$). The effect of passage of time in this study was examined using the analysis of covariance (ANCOVA) in Table S2 (Supplementary file). During the pre-test and post-test phases, behavioral data and brain activity were measured. Training phase consisted on four repetitions of a long task period (15 min) with a rest period (1 min). In the other phases, a short task (80 s) was repeated 30 times with a 5-s rest period. Note that the actual time period for performing the visuomotor task was 0-40 s in the short task (80 s) to be able to obtain a waveform of hemodynamic response function (HRF) (Supplementary file, Fig. S1.A). Therefore, the behavioral data and brain activity were measured at 0-40 s and 0-80 s in each short task, respectively.

### Measurement

#### Behavioral data

The horizontal positions of the target *t*(*i*) and the cursor *c*(*i*) were measured in each task repetition, respectively. Both *t*(*i*) and *c*(*i*) were recorded at every *i*th observed point with a sampling frequency of 1000 Hz.

#### Brain activity

Brain activity was measured using a NIRS device (BriteMK2, Artinis Medical Systems, The Netherlands). To investigate the frontal regions, we arranged 18 measurement channels covering those regions. Figure 4C shows the placement of all channels and optodes. Channels located at the middle points between the optodes consisted of transmitters and receivers. To measure prefrontal cortices and motor cortices of both hemispheres, we defined four regions: LF, RF, LM and RM. From the anatomical brain structure, the prefrontal cortex is located in the prefrontal regions while the PMC/SMA and M1 are located in the forward and backward motor regions, respectively. Channels L1—L4, R1—R4, L5—L8, and R5—R8 are arranged in four regions: LF, RF, LM, and RM, respectively. A previous study reported that F3 and F4 located in frontal cortex (superior and middle frontal gyrus), while C3 and C4 are positioned in motor cortex (precentral and postcentral gyrus)^42^. This indicates that regions anterior to F3 and F4, to C3 and C4 are the PFC and the MC, respectively. Thus, some transmitters on LF and RF, and some receivers on LM and RM were arranged in F3, F4, C3, C4 of the international 10-20 system, respectively. Two channels (S1, S2) centered in a short distance between the optodes were considered to reflect blood flow in the scalp. Since the measured brain activity may contain noise related to scalp blood flow during the visuomotor task, we arranged the channels measured at the short distance to eliminate this noise.

### Analysis

#### Analysis procedure

**Fig. 5:**
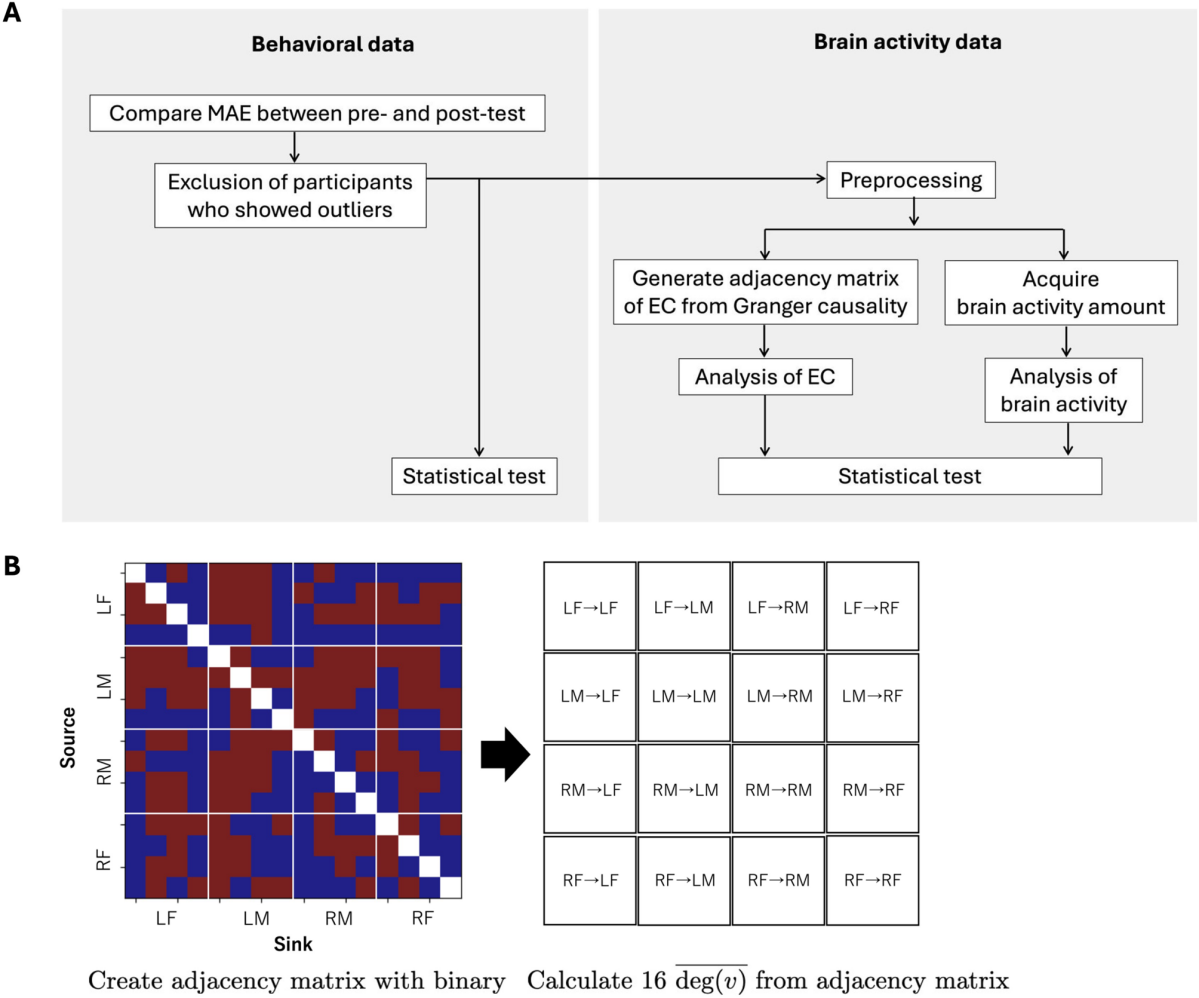
Explanation of analysis. (**A**) Analysis procedure. (**B**) Calculation of 16 average degree, $\overline{\textrm{deg}(v)}$ using an adjacency matrix. Red and blue matrix elements indicate with/without the effective connectivity (EC), respectively. White elements were eliminated from the analysis as the source and sink channels were identical.

Figure 5A shows the analysis procedure. Behavioral data has previously been used to assess brain plasticity, so behavioral data at the pre-test and post-test were examined at first. We calculated the average behavioral data of 30 trials for each participant at the pre-test and post-test, and compared box plots of average behavioral data between pre-test and post-test. As a result, one participant showed an outlier with an interquartile range value > 1.5 from the upper quartiles, so the participant was eliminated from the subsequent analyses. The effect of the outlier was examined using the sensitivity analysis (see Table S1 in Supplementary file). Regarding nine participants included in the analysis, we compared behavior and brain activity data at the pre-test and post-test after processing. We considered possibilities of fatigue and learning effects within 30 trials, and it is not appropriate to compare pre-test with post-test by average data of 30 trials. Thus, 30 trials was divided by three blocks: trials 1–10, 11–20, and 21–30, and a block of pre-test was compared to correspondent block of post-test (i.e., pre-test vs. post-test at trials 1–10, 11–20, 21–30). To compare the block between pre-test and post-test, the *p* value was calculated using Wilcoxon signed-rank test. Although the numerous statistical tests were performed due to the comparison in three blocks and 16 average degree, we did not consider the multiple comparison correction since we formed two hypotheses in advance. One is that there are significant decreases in the brain network of LM$\rightarrow$RF and RF$\rightarrow$LM in the last 10 trials at the post-test compared to in the same block at the pre-test. The other is that there is a significant improvement of the behavior in the first 10 trials at the post-test compared to in the same block at the pre-test. Both hypotheses were formed based on the results of Fig. S2 (supplementary file). The Cohen’s *d* also used to evaluate the effect size. Cohen’s guidelines, *d* = 0.2, 0.5, and 0.8 are interpreted effect sizes as small, medium, or large, respectively. According to this guidelines, differences between pre-test and post-test were evaluated.

#### Behavioral data

We adopted MAE as behavioral data. The MAE between the horizontal positions of target *t*(*i*) and cursor *c*(*i*) measured in each repetition was calculated using the following equation:

2 $$\begin{aligned} MAE= \frac{1}{n} \sum _{i=1}^n |c(i) - t(i)|, \end{aligned}$$

where *i* and *n* represent the index and the total number of points observed in a repetition, respectively. As it is difficult to respond to the task quickly at the beginning of the task and hand fatigue by repeated trials impairs task performance, the time window (TW) of MAE was set at 5-35 s during the task period. A small MAE indicates improvement in motor skills. We acquired 30 MAE values for each participant during a test phase.

### Brain activity data

#### Preprocessing

We used measured oxygenated hemoglobin (oxy-Hb) as brain activity because the increased oxy-Hb is related to cortical activation. First, we reduced physiological noise by discrete cosine transform (DCT) and moving average. Next, we transformed data into Z-scores based on oxy-Hb during the rest period to standardize data. Next, we eliminated oxy-Hb measurements at short distance, which can be considered scalp-related noise, based on a previous study^43^.

#### Brain activity

To assess changes in brain activity, we calculated the integrated value of oxy-Hb during the pre-test and post-test phase. The TW comprised 30-70 s of each phase, in reference to the complementary analysis (see Supplementary file, Fig. S1.A and B). Next, we created color maps consisting of four regions: LF, RF, LM and RM, to visually show differences between post-test and pre-test (post-test minus pre-test). The variation intensity of each channel was indicated by *t* values. For the comparison between post-test and pre-test, a paired *t*-test was used to acquire the *t* value. Finally, we performed the Wilcoxon signed-rank tests to confirm the significant differences of the integrated values between pre-test and post-test at each channel.

#### Effective connectivity (EC)

We performed a Granger causality analysis to investigate the EC between all paired channels. Granger causality is a statistical method to analyze whether there are causal relations between two channels of a time-series signal. Although applying Granger causality to hemodynamic data such as fMRI and NIRS is controversial due to delay of hemodynamic response latency, a previous fMRI study reported the result analyzed by Granger causality was better if there were sufficient data samples and noise was properly removed^44^. Moreover, Granger causality is adopted in many studies using NIRS^20,21,45,46^. To examine the causality between $x_1$ and $x_2$, we model the time-series of $x_1$ in two-ways. One is the vector autoregression (VAR) model, where the future value of $x_1$ is estimated using the past information of both $x_1$ and $x_2$, and the other is the VAR model in which $x_1$ is estimated using the past information itself. These models can be represented by the following equations,

3 $$\begin{aligned} x_1(t)= & \sum _{i=1}^m a_{11,i} \cdot x_1(t-i) + a_{12,i} \cdot x_2(t-i) + \epsilon (t), \end{aligned}$$

4 $$\begin{aligned} x_1(t)= & \sum _{i=1}^m a^\prime _{11,i} \cdot x_1(t-i) + \epsilon ^\prime (t) , \end{aligned}$$

where, *a* and *m* represent the coefficients and order of the VAR model, respectively. $\epsilon$ corresponds to the residual error. If the variances of $\epsilon (t)$ and $\epsilon ^\prime (t)$ are compared using the *F*-test, and $\epsilon (t)$ is significantly small, it is considered that $x_2$ causes $x_1$, which means connectivity of $x_2 \rightarrow x_1$. To complete the Granger causality analysis between all paired channels, we generated an adjacency matrix with *p* values from *F*-test. If connectivity had *p* < 0.05, it was defined that EC was confirmed. This way, the values of adjacency matrix were binarized, and the adjacency matrix of EC generated for each phase. Finally, from the adjacency matrix of EC, $\overline{\textrm{deg}(v)}$ was calculated using the following equation:

5 $$\begin{aligned} \overline{\textrm{deg}(v)} = \frac{\sum _{i=1}^N d(v_i)}{N-1}, \end{aligned}$$

where $v_i$ represents the $i^{\textrm{th}}$ channel, and $d(v_i)$ is the degree of the $i^{\textrm{th}}$ channel. We examined $\overline{\textrm{deg}(v)}$ differences in four regions: LF, RF, LM and RM at pre-test and post-test. Figure 5B shows the adjacency matrix of EC and 16 $\overline{\textrm{deg}(v)}$ between two regions. The left of the figure is the adjacency matrix of the EC, and vertical and horizontal represent the source channels and the sink channels, respectively. Both source channels and sink channels are classified into four regions: LF, RF, LM and RM, so 16 $\overline{\textrm{deg}(v)}$ were acquired from the adjacency matrix of EC, as shown in the right figure. Next, we compared 16 $\overline{\textrm{deg}(v)}$ during the pre-test and post-test using a Wilcoxon signed-rank test. When $\overline{\textrm{deg}(v)}$ at the post-test is smaller than at the pre-test, we evaluated that the brain network declines with motor training (Fig. 5).

## Supplementary Information

Supplementary Information.

## Data Availability

Datasets generated for this study are available upon request from the corresponding author.
