# Supplementary material for: Brain network and activity in frontal regions change in the process of visuomotor adaptation
Source: Sci Rep. 2025 Dec 11;15:43649. doi: 10.1038/s41598-025-27589-3 (PMC12698721; doi:10.1038/s41598-025-27589-3)
Supplement: Supplementary file 1 — Supplementary Information. [file 41598_2025_27589_MOESM1_ESM.pdf]

Supplementary information for

Brain Network and Activity in Frontal Regions Change in the Process of Visuomotor Adaptation

Saki Niiyama, Rie Yoshida, Megumi Miyashita, and Toshiyuki Kondo

The time window for brain activity

To determine the appropriate time window (TW) for brain activity analysis, we obtained the variation in brain activity at each candidate TW with a time width of 40 s, between the pre-test and post-test, then compared the p value using the Wilcoxon signed-rank test. After the selection of participants based on the exclusion criterion for the analysis, the Wilcoxon signed-rank tests were performed to the average brain activity across all analyzed participants. Figure S1.B represents the transition of the p value at each candidate TW. In TW: 30-70 s, the greatest number of channels (L6, L7, R3, and R5) that showed relatively large changes between pre-test and post-test $(p < 0.1)$ were confirmed. We also examined the time-series of the total average measured oxy-Hb (Fig. S1.A). In principle, the HRF model indicates that brain activity is the plateau in TW: 20-60 s, which is the middle of the short task period. However, the observed plateau was later than the HRF model. Based on the evidence, we used 30-70 s as TW for the analysis.

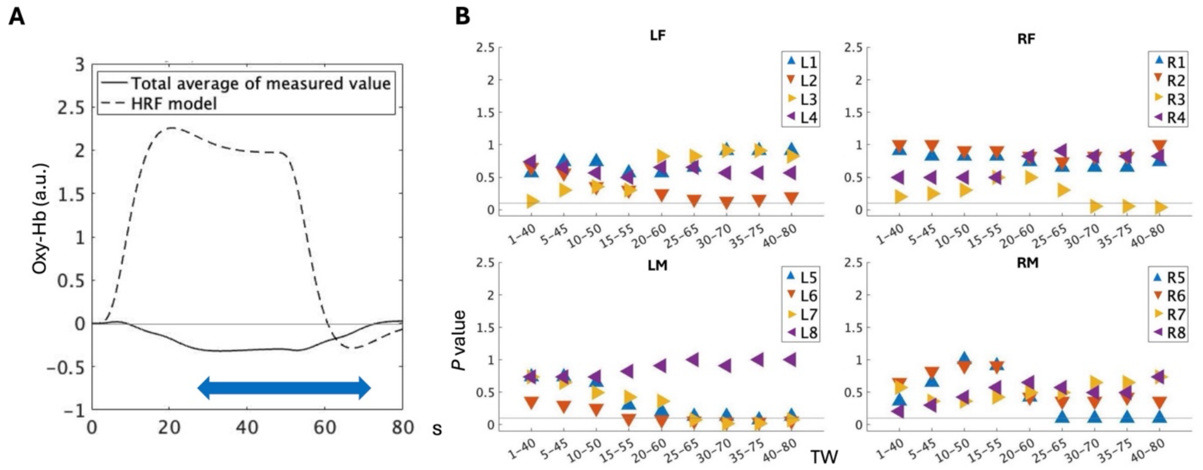

Fig. S1. Pre-analyses for determining the appropriate TW used to calculate brain activity ($n = 9$). A: Time-series graphs of measured oxy-Hb and HRF model. B: Transition of the p value using the Wilcoxon signed-rank test at each TW. Each colored triangle represents the channel measured by NIRS, and the horizontal line indicates $p = 0.1$.

The sensitivity analysis

To investigate the relation of the brain network between the prefrontal cortex (PFC) and motor cortex (MC), and the behavior, we compared the pre-test and post-test using the Wilcoxon signed-rank test, from the perspectives of the brain network and behavior in the paper. Results showed the significant behavioral improvement in the first 10 trials, and the significant decrease of brain network (PFC-MC) in the last 10 trials, of all 30 trials at the post-test, compared to the same block of trials at the pre-test.

Although the statistical test above was performed without the participant who showed the outlier, we also performed the sensitivity analysis to consider the effect of outlier. As the sensitivity analysis, the Wilcoxon signed-rank test was performed without the participant exclusion (i.e., with all participants) in order to compare the pre-test and post-test in the same trial block (behavior: the first 10 trials, brain network: the last 10 trials). Table S1 shows results of the behavior and brain network without the participant exclusion. The result showed that LM → RF, RF → LM in the last 10 trials at the post-test decreases significantly compared to the pre-test (LM → RF: $p(W) = 0.01(43.5), d = 1.28$, RF → LM: $p(W) = 0.03(48.5), d = 0.86$), which indicated same outcome analyzed by the sample size of the participant exclusion. However, the behavioral result showed no significant improvement in the first 10 trials at the post-test compared to the pre-test ($p(W) = 0.16(42), d = 0.04$), and did not match the outcome analyzed by the sample size of the participant exclusion. The sensitivity analysis suggests that the outlier influences on the main outcome of behavior. It is considered that the behavioral improvement is affected by the fatigues of the arm and vision in the experiment day including the variation of brain network through motor learning. The fatigue is considered to related to the factor of outlier.

**Behavior**			Trials 1-10				
			Pre-test		Post-test			
			Mean	SD	Mean	SD	$p(W)$	d
			2.87	0.61	2.81	1.61	0.16 (42)	0.04

**Brain network**			Trials 21-30					
Region			Pre-test		Post-test			
Source		Sink	Mean	SD	Mean	SD	$p(W)$	d
LF	➡	LF	2.07	0.52	1.73	0.84	0.45 (35.5)	0.28
	➡	LM	3.20	0.97	2.27	0.70	0.05 (32)	0.74
	➡	RF	2.90	0.80	2.57	0.70	0.45 (24)	0.26
	➡	RM	2.57	0.80	2.83	0.91	0.50 (16.5)	-0.17
LM	➡	LF	3.03	1.05	2.53	0.92	0.47 (19)	0.35
	➡	LM	2.00	0.52	1.70	0.84	0.34 (25.5)	0.26
	➡	RF	3.10	0.67	1.77	0.72	0.01 (43.5)*	1.28
	➡	RM	2.57	0.93	3.20	1.01	0.23 (12)	-0.40
RF	➡	LF	3.13	0.77	2.83	0.57	0.86 (15.5)	0.25
	➡	LM	3.03	0.79	1.97	0.82	0.03 (48.5)*	0.86
	➡	RF	1.67	0.97	1.73	0.70	0.82 (16)	-0.05
	➡	RM	2.47	1.08	3.00	0.54	0.33 (17.5)	-0.36
RM	➡	LF	2.53	0.77	2.57	0.75	0.89 (25.5)	-0.03
	➡	LM	2.57	1.18	2.97	0.78	0.82 (25)	-0.23
	➡	RF	2.43	1.09	3.00	0.77	0.36 (14)	-0.38
	➡	RM	1.87	1.25	2.73	0.60	0.09 (10.5)	-0.64

Table S1. Results of the behavior and brain network without the participant exclusion ($n = 10$). The behavior and brain network were analyzed by mean absolute value (MAE) and average degree, $\overline{\deg(v)}$, respectively. The p values were calculated by Wilcoxon signed-rank test, and values in bracket represent statistics, W ($* p < 0.05$). The d value represents Cohen's effect size; SD represents the standard deviation.

Behavior	Trials 1-10									
					ANOVA		ANCOVA			
	Pre-test		Post-test				Phase		Interaction	
	Mean	SD	Mean	SD	$F(1,8)$	p	$F(1,7)$	p	$F(1,7)$	p
	2.80	0.60	2.32	0.41	8.82	0.02*	0.04	0.85	0.50	0.50

Brain network		Trials 21-30									
						ANOVA		ANCOVA			
Channel		Pre-test		Post-test				Phase		Interaction	
Source	Sink	Mean	SD	Mean	SD	$F(1,8)$	p	$F(1,7)$	p	$F(1,7)$	p
LF	→ LF	2.07	0.55	1.85	0.80	0.31	0.59	1.85	0.22	2.21	0.18
	→ LM	3.30	0.98	2.33	0.71	4.66	0.06	1.03	0.34	2.07	0.19
	→ RF	2.96	0.82	2.59	0.74	0.67	0.44	0.34	0.58	0.53	0.49
	→ RM	2.59	0.85	3.00	0.78	0.61	0.46	7.95	0.03*	7.18	0.03*
LM	→ LF	3.11	1.08	2.63	0.92	0.89	0.37	0.63	0.45	0.95	0.36
	→ LM	1.96	0.54	1.70	0.89	0.40	0.54	2.80	0.14	3.34	0.11
	→ RF	3.11	0.71	1.74	0.76	14.01	0.01*	0.003	0.96	0.48	0.51
	→ RM	2.63	0.96	3.22	1.07	1.13	0.32	0.07	0.80	0.01	0.93
RF	→ LF	3.22	0.76	2.85	0.60	0.76	0.41	1.59	0.25	2.09	0.19
	→ LM	3.07	0.83	1.85	0.78	9.13	0.02*	0.10	0.76	0.03	0.86
	→ RF	1.70	1.02	1.78	0.73	0.02	0.88	1.16	0.32	1.14	0.32
	→ RM	2.56	1.11	3.00	0.58	0.76	0.41	1.51	0.26	1.20	0.31
RM	→ LF	2.63	0.75	2.74	0.55	0.10	0.76	1.35	0.28	1.26	0.30
	→ LM	2.59	1.24	3.00	0.82	0.43	0.53	0.11	0.75	0.20	0.67
	→ RF	2.56	1.08	3.07	0.78	0.98	0.35	0.61	0.46	0.39	0.55
	→ RM	2.00	1.25	2.67	0.60	2.48	0.15	3.65	0.10	2.67	0.15

Table S2. Results of the ANOVA and the ANCOVA ($n = 9$). The behavior and brain network were analyzed by mean absolute error (MAE) and average degree ($\overline{\deg(v)}$), respectively ($* p < 0.05$). Mean and standard deviation (SD) are values without the adjustment of the interval in days. The phase in the table represents the effect of phase (pre-test and post-test), and the interaction represents the synergistic effect of the phase and interval in days between the pre-test and post-test (interval in days: $n = 9$, $\text{mean} \pm \text{standard deviation} = 2.7 \pm 0.5$, $\text{median} = 3$).

The effect of the interval in days

To examine the effect of the interval in days between the pre-test and post-test, we performed the analysis of covariance (ANCOVA) with the participant exclusion ($n = 9$). We also performed the analysis of variance (ANOVA) to compare the result of Wilcoxon signed-rank test (primary adopted method in the paper) since the ANCOVA is the parametric method and is not appropriate for small sample sizes that cannot ensure the normality. Both tests were performed to compare the pre-test and post-test in the same trial block in a similarly to the comparison of the sensitivity analysis above (behavior: the first 10 trials, brain network: the last 10 trials). Table S2 represents the comparison between the pre-test and post-test, of the brain network in the last 10 trials and of the behavior in the first 10trials respectively, using the ANCOVA and the ANOVA. The behavioral result showed that the improvement in the first 10 trials at the post-test was great, compared to the pre-test ($F(1,8) = 8.82,\ p = 0.02$), as well as the result of the Wilcoxon signed-rank test. However, the effect of phase (pre-test and post-test) and the interaction effect of the phase and interval in days were not observed under the adjustment by the interval in days (the phase: $F(1,7) = 0.04,\ p = 0.85$, the interaction: $F(1,7) = 0.50,\ p = 0.50$). The result of ANOVA in brain network showed the same outcome (decrease at the post-test compared to the pre-test) with the Wilcoxon signed-rank test in LM → RF,

RF → LM (LM → RF: $F(1,8) = 14.01$, $p = 0.01$, RF → LM: $F(1,8) = 9.13$, $p = 0.02$), but there were no decrease after the adjustment of the interval in days (LM → RF: the phase: $F(1,7) = 0.003$, $p = 0.96$, the interaction: $F(1,7) = 0.48$, $p = 0.51$, RF → LM: the phase: $F(1,7) = 0.10$, $p = 0.76$, the interaction: $F(1,7) = 0.03$, $p = 0.86$). The ANCOVA with the sample size of the participant exclusion revealed that the interval in days was related to brain plasticity.

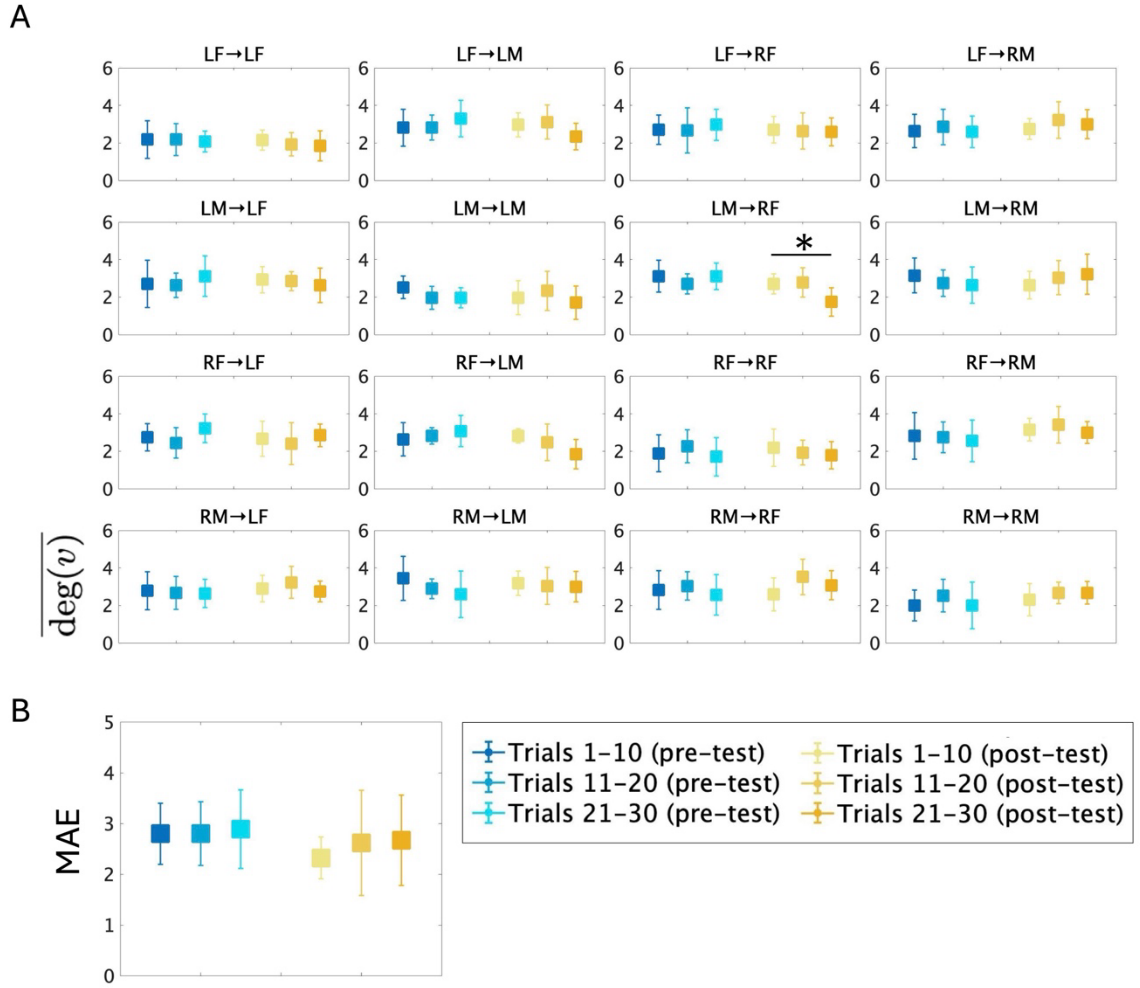

Fig. S2. Comparisons of intra-phases ($n = 9$). The square and bar represent the mean and standard deviation, respectively. A: Brain network, B: Behavior. The behavior and brain network were analyzed by mean absolute error (MAE) and average degree ($\overline{\deg(v)}$), respectively ($*$ false discovery rate (FDR) < 0.05).

The transition of effect across blocks of trials

To compare the variation of intra-phase (trials 1-10, 11-20, and 21-30 at the pre-test or post-test), the Friedman tests were performed to the behavior and brain network of each phase at first, and the post-hoc test (Wilcoxon signed-rank test) was performed if the Friedman test showed the $p < 0.05$. The p value of post-hoc test was corrected by the false discovery rate (FDR)< 0.05. Figure S2 represents the comparison of intra-phase. Behavior showed no significant differences, whereas the brain network (LM → RF) at the post-test decreased in trials 21-30 compared to trials 1-10.

Based on results of Fig. S2, we formulated two hypotheses: one related to the brain network and the other to the behavior. The former hypothesis is that there are significant decreases of brain network of LM→RF and RF→LM in the last 10 trials at the post-test compared to in the same block at the pre-test, since two effective connectivity (EC), LM→RF, RF→LM, in trials 21-30 at the post-test were visually observed to decrease compared in the same block at the pre-test in Fig. S2.A. The latter is that there is a significant improvement of the behavior in the first 10 trials at the post-test compared to in the same block at the pre-test, from the evidence that the mean absolute error (MAE) in trials 1-10 at the post-test improved compared to in the same block at the pre-test in Fig. S2.B. Therefore, statistical tests for the comparison between the pre-test and post-test were performed under following conditions: only on two EC of the last 10 trials in the brain network and on the first 10 trials in the behavior.
